# Mechanism of pyocyanin abolishment caused by *mvaT mvaU* double knockout in *Pseudomonas aeruginosa* PAO1

**DOI:** 10.1080/21505594.2019.1708052

**Published:** 2019-12-28

**Authors:** Limin Dong, Jing Pang, Xiukun Wang, Youwen Zhang, Guoqing Li, Xinxin Hu, Xinyi Yang, Chung-Dar Lu, Congran Li, Xuefu You

**Affiliations:** a Beijing Key Laboratory of Antimicrobial Agents, Institute of Medicinal Biotechnology, Chinese Academy of Medical Sciences & Peking Union Medical College, Beijing, China; b Department of Biomedical and Nutritional Sciences, University of Massachusetts at Lowell, Lowell, MA, USA

**Keywords:** Pyocyanin, *mvaT*, *mvaU*, H-NS, quorum sensing

## Abstract

MvaT and MvaU are global transcriptional regulators belonging to the H-NS family, and pyocyanin is an important virulence factor produced by *Pseudomonas aeruginosa. mvaT mvaU* double knockout mutant of *P. aeruginosa* PAO1 demonstrated pyocyanin abolishment in the previous study. Here, we further explored the mechanism. Two main directions were studied: pyocyanin biosynthesis pathway and QS system. The effect on the expression of the pyocyanin biosynthesis genes was evaluated by promoter strength determination and Real-Time PCR assay, and significant changes leading to low pyocyanin production were found. The effect on the QS system was studied by signal molecule quantification using LC-MS/MS and related gene expression measurements using Real-Time PCR. In *mvaT mvaU* double knockout, the production of 3-oxo-C12-HSL obviously increased, while those of C4-HSL and PQS obviously decreased, and the changes can be recovered by *mvaT* or *mvaU* complementation. The expressions of transcriptional activator genes binding with QS system signal molecules were all decreased, resulting in decreased formation of signal-transcriptional activator complexes. And the decreased expression of *rhlR* and *pqsE* also led to the lower expression of *phzA1* and *phzA2*. Further exploration found that QS system downregulation may be related to QsrO, a QS system repressor, which was highly upregulated with *mvaT mvaU* double knockout. Hence, the synthesis of pyocyanin was suffocated and the biofilm formation ability was decreased. These results were also confirmed by transcriptome analysis, which demonstrated similar gene expression changes of the aforementioned genes together with decreased expression of other virulence factor genes regulated by QS system.

## Introduction

*Pseudomonas aeruginosa* (*P. aeruginosa*) is an aerobic Gram-negative bacterium that can cause both community-acquired and hospital-acquired infections, posing a particular threat to cystic fibrosis patients, traumatic burn victims, patients with implanted medical devices and immunocompromised individuals [1–3]. *P. aeruginosa* is formidable because of the intrinsic ability to develop antibiotic resistance, formation of impenetrable biofilms and releasing a large number of virulence factors [4]. Pyocyanin (PYO) is a redox-active virulence factor produced by *P. aeruginosa* that can easily penetrate biological membranes. This secondary metabolite helps *P. aeruginosa* accept and transport electrons produced in respiration so the bacteria can survive under oxygen-poor conditions [5]. Pyocyanin has been shown to induce oxidative stress, affect endothelial cell redox status, and cause loss of porosity in the liver sinusoidal endothelial cells [6,7]. Pyocyanin can increase intracellular levels of reactive oxygen species (ROS) and result in oxidative damage to components of the cell cycle and direct damage to DNA [8,9]. In addition, pyocyanin is associated with a decline in lung function and contributes to the dominance of *P. aeruginosa* in the CF lung [10]. Significant levels of pyocyanin have been detected in sputum sol, ear secretions, wounds, and urine in chronic infections caused by *P. aeruginosa* [11–13]. Moreover, pyocyanin plays a major role in animal models of acute and chronic infection caused by *P. aeruginosa* [14].

**Figure 1.**
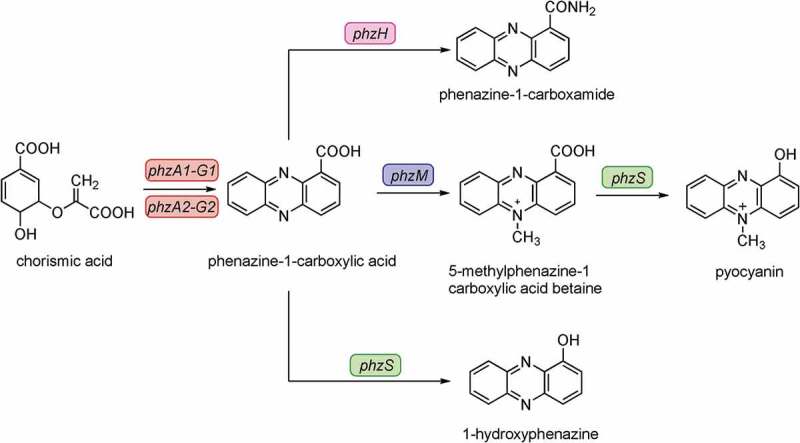
Biosynthesis and signaling system of pyocyanin [15]. Chorismic acid is transformed into phenazine-1-carboxylic acid by the PhzA to G proteins. Then, phenazine-1-carboxylic acid is subsequently converted into different phenazines by the enzymes PhzH, PhzS, and PhzM, respectively. The product of the 5-methylphenazine-carboxylic acid betaine is further transformed into pyocyanin (PYO) by PhzS.

**Figure 2.**
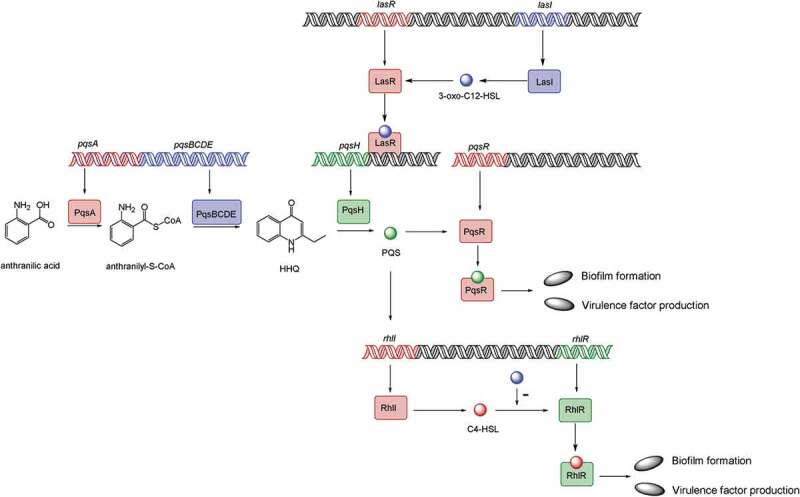
Model of the *P. aeruginosa* quorum-sensing hierarchy. When cells reach a threshold density, the *las* quorum sensing will be induced. LasI directs the synthesis of 3-oxo-C12-HSL, which then binds to and activates LasR. LasR regulates the production of PQS, which is conversed by PqsH from HHQ, catalyzed by PqsA-E. PQS either directly or indirectly induces *rhlI*, which leads to the production of C4-HSL that binds to and activates RhlR. Hence, PQS constitutes a regulatory link between the *las* and *rhl* quorum-sensing system. PQS binds to the transcriptional regulator PqsR to regulate biofilm formation and virulence factor production. The RhlR–C4-HSL complex can induce genes controlled by the *rhl* quorum-sensing system, such as biofilm formation and virulence factor production. 3-oxo-C12-HSL has an inhibitory effect on the association between RhlR and C4-HSL.

Pyocyanin is synthesized through a series of complex steps mediated by gene products encoded by two *phzABCDEFG* operons and the *phzH, phzM, phzS* genes. In the pyocyanin synthetic pathway, chorismic could be transformed into phenazine-1-carboxylic acid by the PhzA-G proteins firstly. Subsequently, phenazine-1-carboxylic acid could be converted to pyocyanin by PhzM and PhzS (Figure 1) [15]. The synthesis is regulated by quorum sensing (QS), which involves in cell-density-dependent accumulation of signal molecules that enable bacteria to modulate the expression of virulence genes [16–18] (Figure 2) and can be repressed by *qsrO* [19]. Several independent studies have revealed that other gene mutations could also influence the synthesis of pyocyanin, such as *kinB* [20], *gbuA* [21], *gacA-gacS* and *vfr* [16]. MvaT and MvaU are global transcriptional regulators belonging to the H-NS family of *P. aeruginosa*, binding the same chromosomal regions, and coregulating the expression of about 350 target genes [22]. Pyocyanin synthesis was induced in the *mvaT mvaU* single mutants but was completely abolished in the double knockout mutant [23].

In this study, we aim to demonstrate the mechanism of pyocyanin abolishment in *P. aeruginosa* PAO1 *mvaT mvaU* double knockout mutant. First, we compared the virulence of *mvaT mvaU* double mutant to the parental strain and the single knockout mutants by a mouse systemic infection model and biofilm formation assay. Then, the effects of *mvaT mvaU* double knockout on the pyocyanin synthesis genes and the quorum-sensing system were studied using promoter strength determination, Real-Time PCR and LC-MS/MS. The results were also confirmed by transcriptome analysis.

## Materials and methods

### Bacterial strains and culture conditions

Strains (from the CAMS Collection Center of Pathogen Microorganisms, CAMS-CCPM-A) and plasmids used in this study are listed in Table S1. *P. aeruginosa* PAO1 wild type strain and *mvaT mvaU* single or double knockout mutants were routinely cultured in Luria-Bertani (LB) broth or on LB agar plates.

### Construction of mutant strains and plasmids

Primers used in this study are listed in Table S2. *mvaT mvaU* single or double knockout mutants were generated using an allelic exchange as previously described [23]. The authenticity of the mutants was confirmed by PCR, RT-PCR and gene sequencing. For complementation of *mvaT* and *mvaU* genes, plasmids pUCP-T and pUCP-U carrying full lengths of *mvaT* or *mvaU* were constructed as before [23] and conﬁrmed by DNA sequence analysis.

### Pyocyanin quantitation assay

Pyocyanin was extracted and quantified from *P. aeruginosa* PAO1 and the *mvaT mvaU* single or double knockout mutants as previously described [24]. Pyocyanin was extracted with 3 mL chloroform from 4 mL cells cultures grown at 37°C for 24 h in glycerol-alanine medium and then reextracted into 2 mL of 0.2 M HCl. The A_520_ of the resulting solution was measured and the concentration of pyocyanin was determined using an extinction coefficient of 2460 M^－1^ cm^－1^. The experiments were performed in triplicate on different days.

### β-galactosidase activity assays

DNA fragments containing the regulatory regions of *phzA1-G1, phzA2-G2, phzH, phzM*, or *phzS* were ampliﬁed by PCR from the genomic DNA of strain PAO1 with Prime STAR polymerase and the primers listed in Table S2. The PCR products were puriﬁed and ligated into pQF50, a broad-host-range *lacZ* transcriptional fusion vector. The nucleotide sequences of the resulting constructs were veriﬁed by nucleotide sequence determination. The β-galactosidase activity was measured with o-Nitrophenyl beta-D-galactopyranoside (ONPG) as the substrate according to procedures from the protocol of Bacterial Adenylate Cyclase Two-Hybrid System Kit, EUROMEDEX. Briefly, bacterial cells were grown in LB broth in the presence of 0.5 mM IPTG and 150 μg/mL of carbenicillin at 30°C till OD_600_ ≈ 0.3. After 30 μL of chloroform and 30 μL of 0.1% SDS solution were added to 2.5 mL of cell suspensions, the cultures were vigorously agitated in a shaker at 37°C for 40 min. 0.1 mL of the cells was added to 0.9 mL of PM2 buffer (70 mM Na_2_HPO_4_.12H_2_O, 30 mM NaH_2_PO_4_ H_2_O, 1 mM MgSO_4_, 0.2 mM MnSO_4_, pH 7.0, add 100 mM β-mercaptoethanol just before use) and were placed in a water bath at 28°C for 5 min. The enzymatic reaction was started by adding 0.25 mL of the ONPG substrate solution and stopped by 0.5 mL of the 1 M Na_2_CO_3_. The OD_420_ was recorded for calculating the enzymatic activities with the correction of bacterial cell absorbance by OD_600_. The experiments were performed in triplicate on different days.

### In vivo infection evaluation

All mice (ICR, female, 18–20 g) were obtained from Beijing Vital River Laboratory Animal Technology Co. Ltd. Mice were infected intraperitoneally with 0.5 mL bacterial suspensions of *P. aeruginosa* PAO1 or *mvaT mvaU* single/double knockouts in 5% mucin. The experiments were performed in triplicate on different days. The animal husbandry and experiments were performed according to national standards of laboratory animals in China (GB/T 35892–2018) [25]. A log-rank test was applied to compare the survival distributions of animals infected by different strains.

### Biofilm formation assay

Biofilm quantification assays were performed in microtiter plates using crystal violet staining according to published protocols [26]. Overnight cultures were diluted to 1 ~ 2 × 10^6^ CFU/mL in Brain Heart Infusion (BHI) broth, 200 μL was allocated to each well of a flat 96-well microtiter plate (Corning, 3599) and cultured at 37°C for 24 h. Planktonic cells were removed and wells were washed with physiological saline. 200 μL crystal violet solution (0.1%, v/v) was added to each well and incubated at 37°C for 15 min. Then, crystal violet solution was removed and wells were washed with double-distilled water 3 times. 200 μL of glacial acid (30%, v/v) was added to each well and absorbance was measured at 595 nm using Perkin Elmer 2300 EnSpire Multilabel Plate Reader.

### Relative growth rate assay

The exponential growth rates of the mutant strains were measured in CAMH broth at 37°C by taking optical density at 600 nm (OD_600_) every 4 min in a Bioscreen C reader (Oy Growth Curves Ab Ltd, FP-1100-C). Four independent cultures per strain were grown overnight until saturation. The cultures were 1000-fold diluted and aliquoted into a Bioscreen C plate in duplicate (0.3 mL/well). The growth rates were estimated from the OD_600_ interval between 0.01 and 0.1, where the growth was observed to be exponential. Relative growth rates of the strains were calculated by comparing the growth rates with that of PAO1.

### Gene expression determined by real-time PCR

*P. aeruginosa* PAO1 and *mvaT mvaU* mutants were grown in LB broth at 37°C with shaking (220 rpm) until OD_600_ ≈ 0.4 (in log phase) or OD_600_ ≈ 1.0 (in early stationary phase); then, 1.5 mL of cells was harvested by centrifugation at 4°C. The RNA extraction of bacteria was performed using RNAprep Pure Cell/Bacteria Kit (TIANGEN), and mRNA was reversed to cDNA using FastQuant RT Kit (TIANGEN). The Real-Time PCR reaction mixture (20 μL) contained 10 μL 2 × Power SYBR Green PCR Master Mix (Applied biosystems by Life technologies), 2 μL forward and reverse primer mix (10 μM each, sequences are listed in Table S2), 1 μL template cDNA and 7 μL nuclease-free water. The cycling conditions were 50°C for 2 s, 95°C for 10 min followed by 40 cycles at 95 ^o^C for 15 s and 60°C for 1 min using 7500 Fast Real-Time PCR System (Applied Biosystems ^TM^). The experiments were performed in triplicate on different days.

**Table 1.** Quantitation of pyocyanin in *P. aeruginosa* PAO1 and the knockout mutants.

Strain	Pyocyanin production without or with complementary plasmids		
	No plasmid	pUCP-T	pUCP-U
PAO1	119	76	87
PAO1ΔT	123	158	158
PAO1ΔU	157	122	126
PAO1ΔTΔU	ND	78	49

ND: not detectable.

### Quantitation of quorum-sensing signaling molecules using LC-MS/MS

The quantitation of AHLs (3-oxo-C12-HSL, C4-HSL) and PQS was performed as previously described [27–30]. Briefly, strains were grown at 37°C in LB broth until OD_600_ ≈ 1.0. For extraction of AHLs, samples were centrifuged at 10,000 g in a Thermo Fresco21 tabletop centrifuge for 20 min at 4°C. 0.6 mL of liquid supernatant was extracted with acid ethyl acetate. The organic phase was dried using a vacuum freeze dryer (CHRIST, ALPHA2-4 LD pius), resolubilized by methanol and filtered with millex (0.22 μM, MERCK). For extraction of PQS, 0.5 mL of cultures were mixed with isovolumic methanol, and samples were centrifuged at 10,000 g in a Thermo Fresco21 tabletop centrifuge for 20 min at 4°C. The supernatant was filtered with millex (0.22 μM, MERCK) and used for LC-MS/MS analysis. The following standards were used: N-(3-oxododecanoyl)-L-homoserine lactone (3-oxo-C12-HSL, Sigma Aldrich), N-[(3S)-Tetrahydro-2-oxo-3-furanyl] butanamide (C4-HSL, Cayman Chemical), 2-heptyl-3-hydroxy-4(1H)-quinolone (PQS, Sigma Aldrich). Three samples were performed for each group.

### Transcriptome analysis

The total RNA extraction of PAO1 wild type and *mvaT mvaU* single/double knockouts were performed using RNAprep Pure Cell/Bacteria Kit (TIANGEN) as described in Real-Time PCR assay with cells harvested at OD_600_ ≈ 0.4, and transcriptome analysis was performed by Novogene (Beijing, China). Briefly, the concentration of purified RNA was measured and the integrity was assessed first. A total amount of 3 μg RNA per sample was then used for transcriptome analysis. Sequencing libraries were generated using NEBNext® Ultra™ Directional RNA Library Prep Kit for Illumina® (NEB, USA) and index codes were added to attribute sequences to each sample. After cluster generation, the library preparations were sequenced on an Illumina Hiseq platform and paired-end reads were generated. Then, differential expression was analyzed using the DESeq R package (1.18.0), which provides statistical routines for determining differential expression in digital gene expression data by a model based on the negative binomial distribution. P-values were adjusted using the Benjamini and Hochberg’s approach for controlling the false discovery rate, and genes with an adjusted P-value of <0.05 were assigned as differentially expressed. Three biological repeats were used for each group.

## Results

### Pyocyanin production influenced by mvaT and/or mvaU mutations

To conduct this study, we constructed a new set of *mvaT* and *mvaU* single and double knockout mutants in the wild type strains of PAO1 as described in Materials and Methods. Consistent with the results reported previously [23,31], pyocyanin synthesis was enhanced in *mvaT* and *mvaU* single knockout mutants while it was totally abolished in the double knockout mutant. Furthermore, the observed deficiency of pyocyanin synthesis in the double knockout mutant could be partly complemented by plasmids carrying *mvaT* or *mvaU* genes (Table 1).

### The influence of mvaT and/or mvaU knockouts on phenotypes of P. aeruginosa PAO1

**Figure 3.**
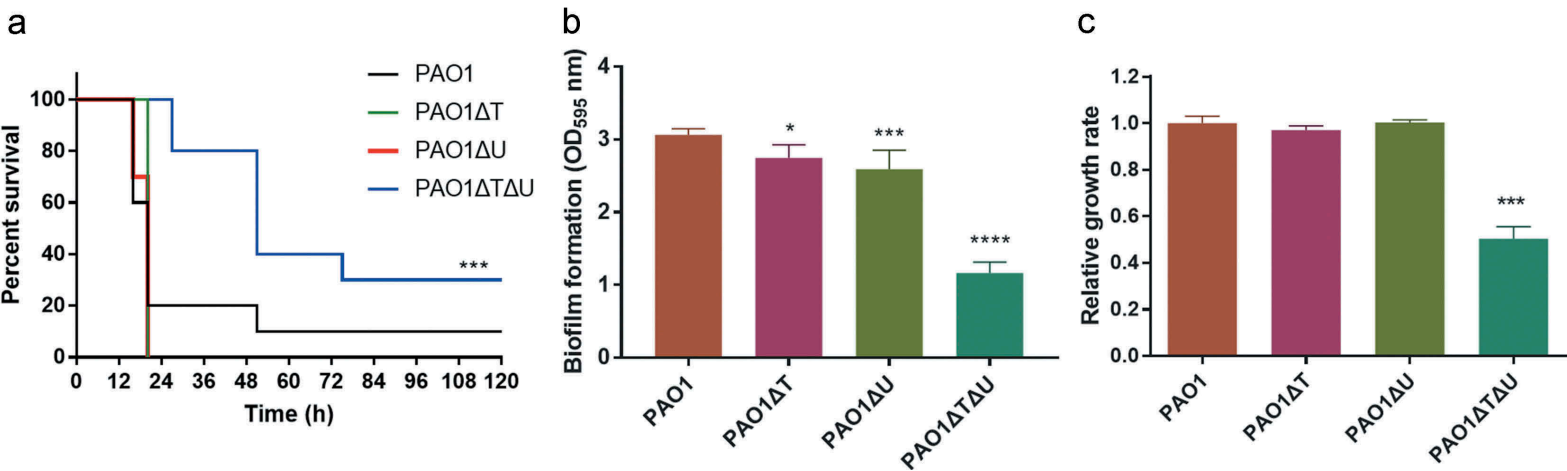
Effect of *mvaT mvaU* knockouts on the phenotype of *P. aeruginosa* PAO1. A: Percent survival of mice infected by PAO1 wild type and *mvaT mvaU* knockout mutants (n = 10), ***P< 0.001 via log-rank test. PAO1: 6.5 × 10^3^ CFU/mice; PAO1ΔT: 4 × 10^3^ CFU/mice; PAO1ΔU: 6.5 × 10^3^ CFU/mice; PAO1ΔTΔU:1.5 × 10^4^ CFU/mice. B: Biofilm formation of PAO1 wild type and *mvaT mvaU* knockout mutants, calculated with one-way ANOVA and Bonferroni’s multiple comparisons (n = 6), *P< 0.05, ***P< 0.001, ****P< 0.0001. C: Relative growth of the *mvaT mvaU* knockout mutants in comparison to PAO1, calculated with one-way ANOVA and Bonferroni’s multiple comparisons (n = 4), ***P< 0.001.

Firstly, we investigated the survival percentages of mice infected by different *P. aeruginosa*. The *mvaT* and *mvaU* single mutants showed hypervirulent features. Mice infected by *mvaT* and *mvaU* single mutants were all died within 20 h. In contrast, the survival rate of mice infected with *mvaT mvaU* double knockout mutation was improved from 10% to 30% compared to PAO1 wild type (Figure 3(a)), suggesting decreased virulence of the double mutant.

Secondly, the biofilm-forming ability was compared. As shown in Figure 3(b), knockout of *mvaT* and/or *mvaU* led to decreased biofilm-forming ability, with that of the *mvaT mvaU* double knockout mutant decreased to 38% of the wild type strain. In addition, the relative growth rates of the mutants were compared to that of the wild type strain, and the double knockout strain demonstrated a growth rate of about two times slower than that of the wildtype strain, while the single knockouts had no obvious changes (Figure 3(c)).

### Effects of mvaT and/or mvaU knockouts on the expression of pyocyanin biosynthesis-related genes

**Figure 4.**
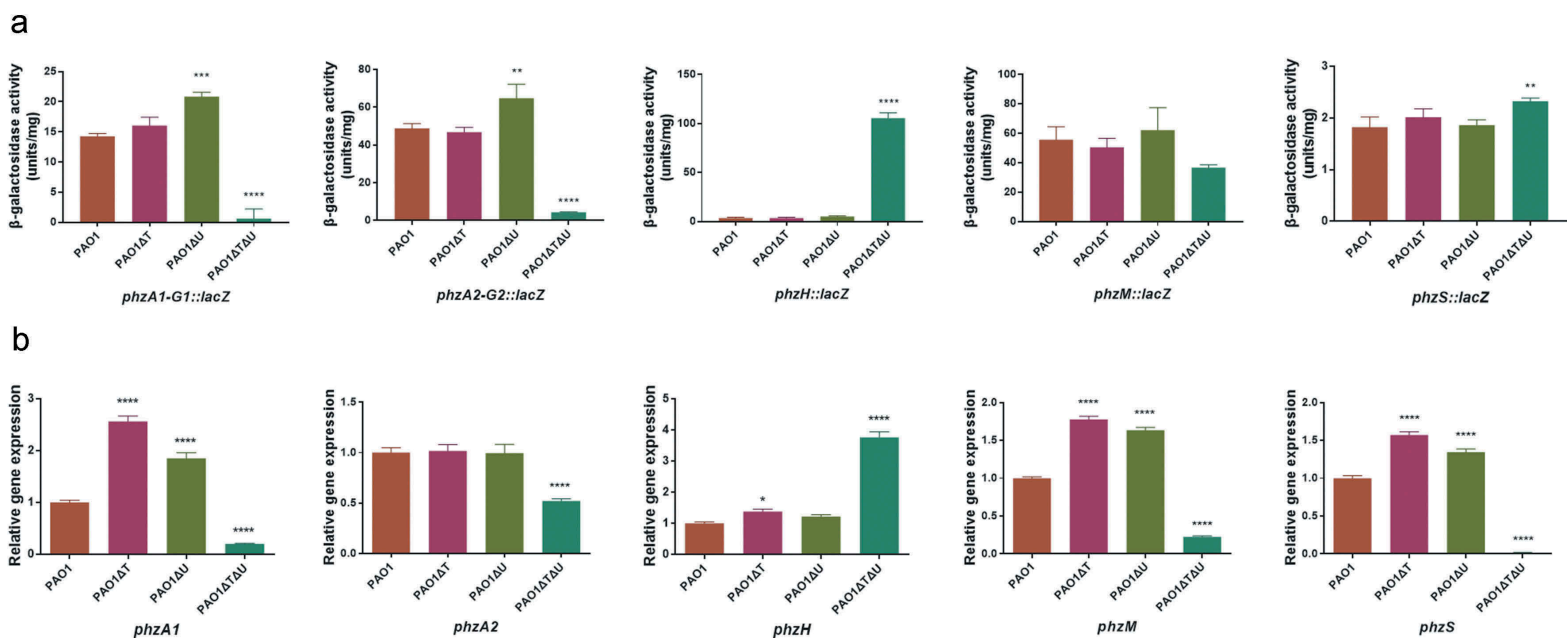
Effect of *mvaT mvaU* knockout mutations on gene expression of pyocyanin biosynthesis system. A: β-galactosidase activity of *phzA-G1/A2-G2/H/M/S: lacZ* fusion covering the regulatory region. B: Relative gene expression of pyocyanin biosynthesis genes detected by Real-Time PCR. Data were calculated with one-way ANOVA and Bonferroni’s multiple comparisons, in comparison to *P. aeruginosa* PAO1, *P< 0.05, **P< 0.01, ***P< 0.001, ****P< 0.0001.

Firstly, the promoter activities of the related genes or operons were compared by measurements of β-galactosidase activities using *lacZ* transcriptional fusion plasmids carrying the promoter regions of the pyocyanin biosynthesis-related genes. As shown in Figure 4(a), the *mvaT* single knockout did not affect the promoter activities of the genes in general, while the promoter activities of *phzA1-G1* and *phzA2-G2* operons were increased 1.21–1.46 times in the *mvaU* single knockout in comparison to the wild type. In the *mvaT mvaU* double knockout mutant, the promoter activities were decreased to 4%-9% for *phzA1-G1* and *phzA2-G2*, and 66% for *phzM*, while those of *phzH* and *phzS* were increased (29.15 times for *phzH*, 1.27 times for *phzS*). Regardless, these changes eventually led to a drastically reduced activity of pyocyanin biosynthesis. Secondly, the transcript levels of pyocyanin synthesis related genes were evaluated by Real-Time PCR. The results (Figure 4(b)) in the log phase showed that the transcript levels of the pyocyanin synthesis-related genes or operons (except for *phzA2*) were generally increased (1.22–2.56 times) in *mvaT* or *mvaU* single knockout mutants in comparison to PAO1. However, in the double knockout mutant, most genes showed decreased expression by RT-PCR (20% for *phzA1*, 52% for *phzA2*, 23% for *phzM*, 1% for *phzS*) except for *phzH* (3.76 times increased), resulting in a lowered level of pyocyanin production. In early stationary phase, the transcript levels of these genes showed similar trends as in the log phase (Fig S4).

### Effects of mvaT and/or mvaU knockouts on the quorum-sensing system

It was well established that pyocyanin synthesis is regulated by the QS system [16–18], a global level of gene regulation that involves intercellular communication by means of cell-density dependent signal molecules. Hence, we conducted experiments to measure the expression levels of genes coding signal molecule catalyzing enzymes (*pqsE, lasI, pqsH,* and *rhlI*), the level of AHLs and PQS compounds, and the expression levels of genes coding for transcriptional activators binding with signal molecules (*lasR, pqsR*, and *rhlR*). We also measured the expression level of *qsrO*, a regulator in the QS system which can down-regulate all QS system regulatory and target genes [19].

**Figure 5.**
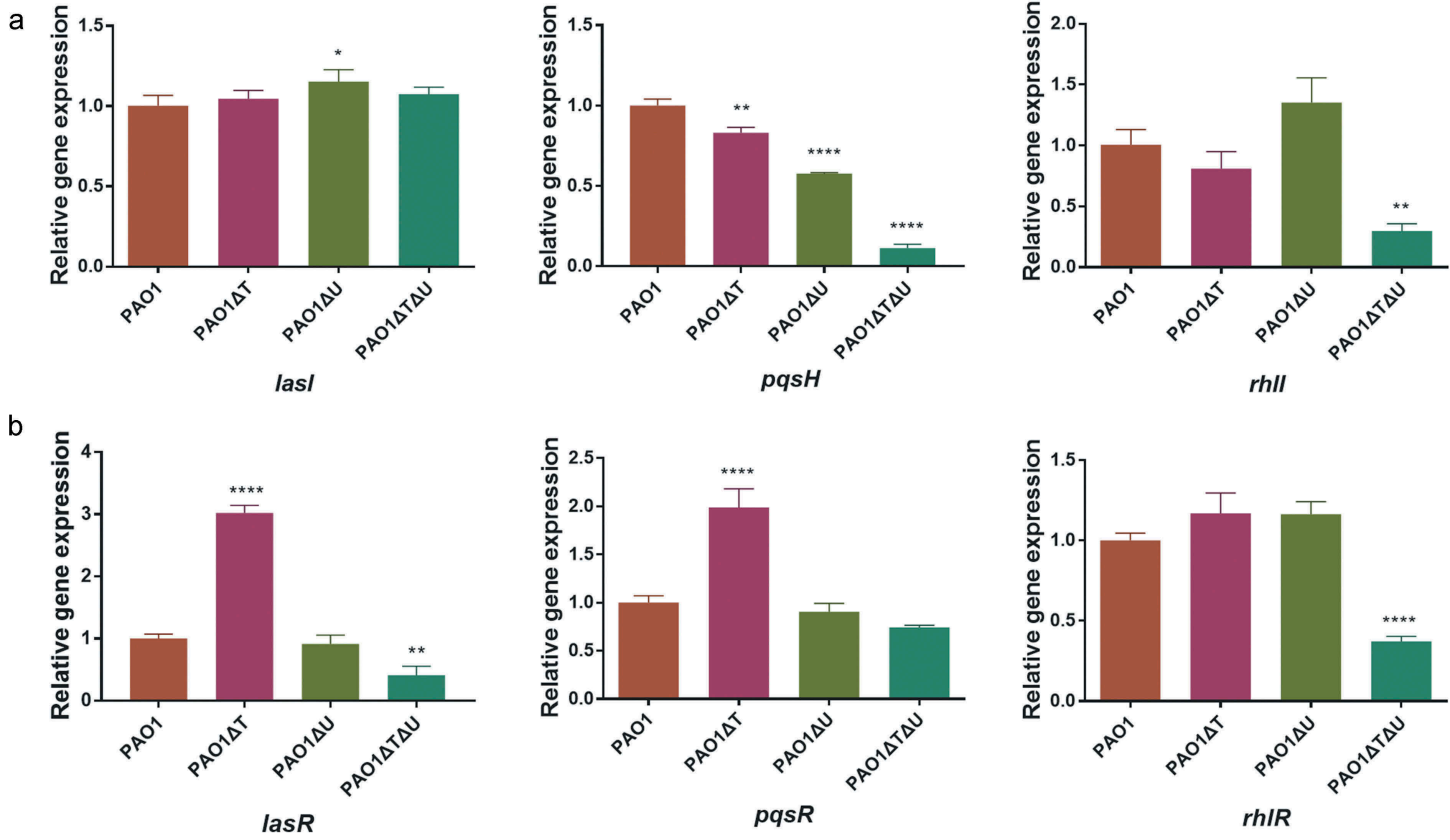
Effect of *mvaT mvaU* knockout mutations on the expression of genes involved in QS system determined by Real-Time PCR. A: Relative expressions of genes coding QS signal molecule synthetase. B: Relative expressions of genes coding transcriptional activator proteins binding with signal molecules. Data were calculated with one-way ANOVA and Bonferroni’s multiple comparisons, *P< 0.05, **P< 0.01, ***P< 0.001, ****P< 0.0001.

The Real-Time PCR results showed that in the *mvaT* single knockout mutant the levels of *lasI, lasR, pqsR* and *rhlR* expression were increased to 1.05–3.02 times, while those of *pqsH* and *rhlI* were decreased (83% for *pqsH*, 81% for *rhlI*). In the *mvaU* single knockout mutant, generally no obvious changes were seen, except for *lasI* and *pqsH*. In the *mvaT mvaU* double knockout, all these genes exhibited lower levels of expression (11%-74%) except *lasI* (Figure 5).

The level of *qsrO* expression increased 23.8 times in the *mvaT mvaU* double knockout, 3.18 times in the *mvaT* single knockout mutant, and showed no obvious change in the *mvaU* knockout mutant in comparison to those in the wild type strain PAO1 (Figure 7(b)). The expression level of *pqsE* was decreased to 7.8% in the *mvaT mvaU* double knockout mutant and 87% in the *mvaT* single knockout mutant but increased to 1.31 times in the *mvaU* single knockout mutant (Figure 7(b)).

**Figure 6.**
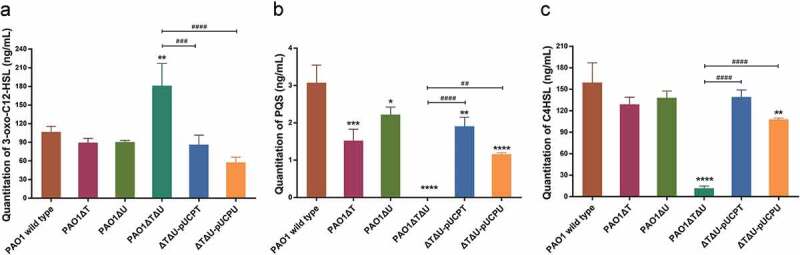
Effect of *mvaT mvaU* knockout mutations on the production of QS system signal molecules AHLs (3-oxo-C12-HSL and C4-HSL) and PQS determined by LC-MS/MS. A: Quantitation of 3-oxo-C12-HSL. B: Quantitation of PQS. C: Quantitation of C4-HSL. Data were calculated with one-way ANOVA and Bonferroni’s multiple comparisons, *P< 0.05, **P< 0.01, ***P< 0.001, ****P< 0.0001.

In addition, we used LC-MS/MS to quantitate the production levels of QS signal molecules AHLs (3-oxo-C12-HSL and C4-HSL) and PQS. For the production of 3-oxo-C12-HSL, no obvious change was observed in single knockouts. However, a significant increase (1.70 times) was observed in the *mvaT mvaU* double knockout mutant. When the deleted genes were complemented by plasmids carrying the corresponding genes, the level of 3-oxo-C12-HSL was decreased to wild type level (Figure 6(a)). The levels of PQS and C4-HSL were significantly decreased (0.5% for PQS, 7% for C4-HSL) by *mvaT mvaU* double knockouts, and were recovered to 38%-62% for PQS and 68%-87% for C4-HSL with the introduction of plasmids carrying *mvaT* or *mvaU*. (Figure 6(b,c)).

Further transcriptome analysis confirmed that in comparison to wild type PAO1 or single knockouts, all genes except *lasI* in the QS system had decreased levels of expression in the *mvaT mvaU* double knockout mutant. This double knockout mutant also decreased the expression of genes for virulence factors and biofilm formation related genes that were regulated by the QS system, such as rhamnolipid and elastase related genes (Table 2).

**Table 2.** List of selected genes whose transcriptions were affected by *mvaT mvaU* mutations.

Gene role and gene ID	Gene name	log_2_Fold Change						Description
		PAO1ΔT	Deviation value	PAO1ΔU	Deviation value	PAO1ΔTΔU	Deviation value	
**Pyocyanin synthesis genes/operons**								
PA4210	*phzA1*			1.3636	0.0254	−1.7608	0.1518	phenazine biosynthesis protein
PA1899	*phzA2*							phenazine biosynthesis protein
PA0051	*phzH*					1.5799	0.0212	phenazine-modifying protein
PA4209	*phzM*	1.1577	0.0429	0.96629	0.0258	−1.8647	0.1073	phenazine-specific methyltransferase
PA4217	*phzS*			0.99553	0.0435	−3.8853	0.3877	hypothetical protein
**Quorum-sensing system genes**								
PA1432	*lasI*					1.1403	0.0210	acyl-homoserine-lactone synthase
PA1002	*phnB*					−2.1815	0.0522	anthranilate synthase component II
PA1430	*lasR*					−0.46348	0.0214	transcriptional regulator LasR
PA1431	*rsaL*					−1.7046	0.1243	regulatory protein RsaL
PA3476	*rhlI*					−2.6876	0.0798	acyl-homoserine-lactone synthase
PA3477	*rhlR*					−3.305	0.0469	transcriptional regulator RhlR
PA0996	*pqsA*					−3.7675	0.0580	anthranilate–CoA ligase
PA0997	*pqsB*					−3.5969	0.0488	hypothetical protein
PA0998	*pqsC*					−3.6549	0.0608	hypothetical protein
PA0999	*pqsD*					−3.5349	0.1121	3-oxoacyl-ACP synthase
PA1000	*pqsE*					−3.0798	0.1166	thioesterase PqsE
PA2587	*pqsH*					−2.402	0.0221	2-heptyl-3-hydroxy-4(1H)-quinolone synthase
PA1003	*mvfR (pqsR)*					−1.2653	0.0219	transcriptional regulator MvfR
PA2226	*qsrO*	2.7971	0.0456			4.5068	0.0216	hypothetical protein, regulator of QS and virulence
**Other virulence factor genes**								
PA3479	*rhlA*					−3.9933	0.0683	rhamnosyltransferase subunit A
PA3478	*rhlB*					−3.3193	0.0284	rhamnosyltransferase subunit B
PA1130	*rhlC*					−2.0056	0.0300	rhamnosyltransferase
PA3724	*lasB*					−5.7022	0.0764	elastase LasB
**Biofilm formation**								
PA2232	*pslB*					−1.5192	0.0215	biofilm formation protein PslB
PA2233	*pslC*					−1.2947	0.0231	biofilm formation protein PslC
PA2234	*pslD*					−1.3012	0.0229	biofilm formation protein PslD
PA2235	*pslE*					−1.4038	0.0215	biofilm formation protein PslE
PA2236	*pslF*					−1.1612	0.0229	biofilm formation protein PslF
PA2237	*pslG*					−0.91282	0.0237	biofilm formation protein PslG
PA2238	*pslH*					−1.126	0.0236	biofilm formation protein PslH
PA2239	*pslI*					−0.99985	0.0237	biofilm formation protein PslI
PA2240	*pslJ*					−0.72316	0.0233	biofilm formation protein PslJ
PA3058	*pelG*					0.59858	0.0357	pellicle/biofilm biosynthesis Wzx-like polysaccharide transporter PelG
PA3706	*wspC*					−0.47283	0.0235	biofilm formation methyltransferase WspC

log_2_Fold Change: log_2_ value of the mutant signal in comparison to PAO1 signal; log_2_Fold Change > 0, upregulated; log_2_Fold Change<0, downregulated.

## Discussion

Pyocyanin production was previously reported to be completely abolished in PAO1 *mvaT mvaU* double mutant [23]. In this study, we aim to reveal the related regulatory mechanism that causes this phenotype. Compared to PAO1 wild type, *mvaT mvaU* double knockout mutant demonstrated lower pathogenicity, lower biofilm formation ability and decreased growth rate. The virulence changes induced by *mvaT mvaU* double knockout were in accordance with the abolishment of pyocyanin production. However, no apparent change in cell morphology can be observed in the *mvaT mvaU* single or double knockout mutants by scanning electron microscopy (SEM) or transmission electron microscopy (TEM) in comparison to the wild type (Fig S3).

Pyocyanin is a secondary metabolite produced by *P. aeruginosa* in the stationary phase, which is synthesized through a series of enzymatic reactions by PhzA-G, PhzM, and PhzS proteins [15]. In *mvaT mvaU* double knockout, the expression of genes for pyocyanin biosynthesis was reduced, which resulted in the abolishment of pyocyanin production. It was noted that the promotor activity of *phzS* was increased while the transcript level of *phzS* was decreased in *mvaT mvaU* double knockout. The discrepancy of *phzS-lacZ* promoter activities and *phzS* transcript measurements by RT-PCR strongly suggested that *phzS* expression is mainly controlled by the distal *phzA1* promoter(s) (downregulated) apart from the proximal *phzS*-only promoter (upregulated) of minor contribution in the growth conditions we tested in this study.

It is well known that pyocyanin synthesis is regulated by the QS system [16–18], and knockout of both *mvaT* and *mvaU* also had an obvious influence on the QS system. In the *mvaT mvaU* double knockout mutant, the production of 3-oxo-C12-HSL was significantly increased, while the transcriptional level of *lasR* was obviously decreased. As a result, the level of LasR/3-oxo-C12-HSL complex, which has been reported to regulate PQS production [32], was decreased. Indeed, in the *mvaT mvaU* double mutant, the transcriptional levels of genes related to PQS syntheses such as *pqsA, pqsBCDE,* and *pqsH* [33,34] were all significantly decreased. Consequently, there was almost no PQS produced in the *mvaT mvaU* double knockout mutant. At the same time, the expression of *pqsR* for a transcriptional activator that binds with PQS to function was slightly decreased. Hence, it is very likely that the PQS/PqsR complex level was decreased to a very low residual level. The PQS system was reported to act as a link between the *las* and *rhl* QS system, and it is able to enhance the transcription of *rhlI* in *P. aeruginosa* [17]. In *mvaT mvaU* double knockout, transcriptional expression of *rhlI* and *rhlR*, and the production of C4-HSL were all significantly decreased, which led to decreased C4-HSL/RhlR complex level. What is more, 3-oxo-C12-HSL was reported to have an inhibitory effect on the *rhl* QS system [17], the free 3-oxo-C12-HSL in the double knockout mutant may block the association between RhlR and C4-HSL, and further lower the level of C4-HSL/RhlR complex. The decreased levels of PQS/PqsR complex and C4-HSL/RhlR complex resulted in the decreased levels of biofilm formation and expression of a variety of virulence factors, such as pyocyanin, elastase, and rhamnolipids. In addition, RhlR and PqsE are both required to induce *phzA1* and *phzA2* [35]. The obviously decreased transcriptional levels of *rhlR* and *pqsE* further led to the decreased expression of *phzA1* and *phzA2* in *mvaT mvaU* double knockout mutant.

**Figure 7.**
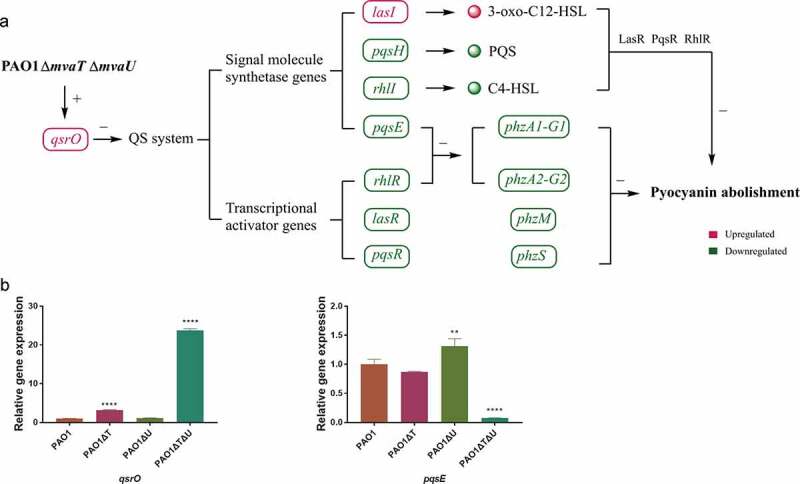
Interactions of *mvaT* and *mvaU* to control biosynthesis and signaling systems of pyocyanin. A: When *mvaT* and *mvaU* were knockout, expression of *qsrO* was significantly increased, which led to the decreased expression of genes coding signal molecule synthetases (*pqsH, rhlI,* and *pqsE*) and transcriptional activators (*rhlR, lasR,* and *pqsR*). The lower expression levels of *phzA1-G1* and *phzA2-G2* caused by decreased expression of *rhlR* and *pqsE*, together with lower expression levels of *phzM, phzS* and the decreased formation of signal-transcriptional activator complexes resulted in pyocyanin abolishment. B: Relative gene expressions of *qsrO* and *pqsE* detected by Real-Time PCR. Data were calculated with one-way ANOVA and Bonferroni’s multiple comparisons, in comparison to *P. aeruginosa* PAO1, **P< 0.01, ****P< 0.0001.

One major issue to justify the hypothesis as described above was that MvaT and MvaU must possess a potential function as transcriptional activators. However, current knowledge of MvaT and MvaU all indicated that these two proteins are transcriptional repressors [22,31,36]. In fact, most genes under the control of MvaT and MvaU were suppressed in the wild type PAO1, and the expression of which was induced in the *mvaT mvaU* double knockout mutant (GSE135506). Few exceptions that display decreased expression include genes in the QS system and pyocyanin synthesis. Therefore, we hypothesized that the downregulation of the QS system by double knockout of *mvaT mvaU* is mediated by another transcriptional repressor that is subjected to direct control by MvaT and MvaU. Further exploration found that QsrO, a repressor of the QS system [19], was significantly upregulated in the *mvaT mvaU* double knockout mutant. Our current hypothesis was that genes repressed in the *mvaT mvaU* double knockout mutant are direct targets of QsrO repressor, a member of the MvaT MvaU regulon. *mvaT mvaU* double knockout results in an increased level of QsrO, which can suppress the expression of the QS system and subsequently genes in the pyocyanin biosynthetic pathway (Figure 7(a)). Future study will be needed to establish the proposed link of MvaT and MvaU to the QS system by QsrO.

In conclusion, this study demonstrated the mechanism of *mvaT mvaU* double knockout on the abolishment of pyocyanin, providing evidence for using *mvaT* and *mvaU* as targets to decrease the pathogenicity or virulence of *P. aeruginosa*.
