# Supplementary material for: Mechanism of pyocyanin abolishment caused by mvaT mvaU double knockout in Pseudomonas aeruginosa PAO1
Source: Virulence. 2019 Dec 28;11(1):57–67. doi: 10.1080/21505594.2019.1708052 (PMC6961724; doi:10.1080/21505594.2019.1708052)
Supplement: Supplemental Material [file kvir-11-01-1708052-s001.pdf]

Supplemental materials

Table S1 Strains and plasmids used in this study

Strain or plasmid	Relevant characteristics	Reference
P. aeruginosa strains		
PAO1	Wild type	ATCC
PAO1-Sm	Spontaneous Sm^r mutant of PAO1	(1)
PAO1ΔT	*mvaT*::Gmr mutant of PAO1	(2)
PAO1ΔU	*mvaU*::Tetr mutant of PAO1	(2)
PAO1ΔTΔU	*mvaT*::Gmr *mvaU*::Tetr mutant of PAO1	(2)
PAO1-pUCPT	Carrying complementation plasmid for *mvaT* gene	(2)
PAO1-pUCPU	Carrying complementation plasmid for *mvaU* gene	(2)
ΔT- pUCPT	Carrying complementation plasmid for *mvaT* gene	(2)
ΔT-pUCPU	Carrying complementation plasmid for *mvaU* gene	(2)
ΔU- pUCPT	Carrying complementation plasmid for *mvaT* gene	(2)
ΔU- pUCPU	Carrying complementation plasmid for *mvaU* gene	(2)
ΔTΔU-pUCPT	Carrying complementation plasmid for *mvaT* gene	(2)
ΔTΔU-- pUCPU	Carrying complementation plasmid for *mvaU* gene	(2)
Plasmids		
pUCP 18	*E. coli*-*P. aeruginosa* shuttle vector	(3)
pUCP-T	Complementation plasmid for *mvaT* gene, derived from pUCP18	(2)
pUCP-U	Complementation plasmid for *mvaU* gene, derived from pUCP18	(2)

Table S2 Primers used in this study

Gene name	Type	Oligonucleotide sequence (5'-3')	Source or reference
Primers for knockout of *mvaT* and *mvaU* genes			
up *mvaT*	Fw	CATAAGCTTCGCGGGCGATCGGGGCGA AAG	(2)
	Rev	ATCGAATTCTTGTCTGTGCTGAGTGGCGGT	
down *mvaT*	Fw	ATCGAATTCACGAAGAACGCCAGCCCAGTG	(2)
	Rev	CATGGATCCGATGTCCGCGCCACCATTGCC	
up *mvaU*	Fw	CCCAAGCTTCAAGGCGATCTTCAAGCCGATCTA	(2)
	Rev	CGGAATTCTTCGTTTCACTCCCGTTCTAAAAA	

down *mvaU*	Fw	CGGAATTCAGCCGGTTTTCCCGACGGCATCCT	(2)
	Rev	CGGGATCCCCGCCATTGTCGCATTCGCGCAGC	
Primers for detection of *mvaT* and *mvaU* genes			
mvaT	Fw	CATGGATCCAGCACAGACAAGGTACCTGAC	(2)
mvaT	Rev	ATCGGATCCTTAGCCGAGCAGGGTGGCCCA	
mvaU	Fw	GGATCCTTTAGAACGGGAGTGAAACGAATG	(2)
mvaU	Rev	GGATCCTTAGCGTTGCAGCCAGGATTCGAC	
Primers for construction of *lacZ* transcriptional fusion plasmids			
up *phzA1*	Fw	CATGCCATGGCCTGTTCCAGAGCCTTT	This study
	Rev	CGGGATCCAGAGGGCTCTCCAGGTAT	
up *phzA2*	Fw	CATGCCATGGCATCGGCCTGCTCAACTGAAT	This study
	Rev	CGGGATCCTGCGAATCTCCGCCAGTTCGAAT	
up *phzH*	Fw	CATGCCATGGTCGAACGTTGCCACGAAATC	This study
	Rev	CGGGATCCAGGGAAACTCCTATAATTG	
up *phzM*	Fw	CATGCCATGGTCATCCCGGGTTTCTTT	This study
	Rev	CGGGATCCCTTTTATTCTCTCTCGTTAC	
up *phzS*	Fw	CATGCCATGGTACCAGACCCACCCGATGT	This study
	Rev	CGGGATCCGGGTGCTTCCTTTTCTCGAGT	
up *rhlR*	Fw	CATGCCATGGTGCAGTAAGCCCTGATCGAT	This study
	Rev	CGGGATCCACGGTGCTGGCATAACAGATA	
up *lasR*	Fw	CATGCCATGGCCGAACTGGAAAAGTGGCTAT	This study
	Rev	CGGGATCCAGCGCTACGTTCTTCTTAAAC	
up *pqsR*	Fw	CATGCCATGGCCCTTATTCCTTTTATTGGG	This study
	Rev	CGGGATCCCAAGGCCGCGGATTCTAAC	
Primers for Real-Time PCR			
oprL	Fw	CCAACAGCGGTGCCGTTGA	
	Rev	GCCATATTGTACTCGCGGGT	
phzA1	Fw	AACGGTCAGCGGTACAGGGAAAC	This study
	Rev	ACGAACAGGCTGTGCCGCTGTAAC	
phzA2	Fw	CTGTAACCGTTCGGCCCCCTTCATG	This study
	Rev	ATGCGAGAGTACCAACGGTTGAAAG	
phzH	Fw	GCTCATCGACAATGCCGAACT	This study
	Rev	GCGGATCTCGCCGAACATCAG	
phzM	Fw	AGCAACCTGGCATTCCACGAG	This study

	Rev	TGCAGGATGGCCTTGGTCAATT	
phzS	Fw	CCGAAGGCAAGTCGCTGGTGA	This study
	Rev	GGTCCCAGTCGGCGAAGAACG	
lasI	Fw	ATGATCGTACAAATTGGTCGGCGCG	This study
	Rev	CGCTCCTTGAACACTTGAG	
lasR	Fw	CTGTGGATGCTCAAGGACTAC	(4)
	Rev	AACTGGTCTTGCCGATGG	
rhlI	Fw	CGGCATCAGGTCTTCATCG	(4)
	Rev	GTAGCGGGTTTGCGGATG	
rhlR	Fw	CGGTCTGCCTGAGCCATC	(4)
	Rev	GCCAGCGTCTTGTTCGG	
pqsA	Fw	GACCGGCTGTATTCGATTC	(4)
	Rev	GCTGAACCAGGGAAAGAAC	
pqsR	Fw	ATCGACGAGGAACTGAAGA	(4)
	Rev	CTGATCTGCCGGTAATTGG	
pqsH	Fw	GCGCGGATCGAGTTCATC	(5)
	Rev	CAGGGCGATTCCCACTGA	
pqsE	Fw	GGATGCCGAATTGGTTTG	This study
	Rev	GGTCGTAGTGCTTGTGGG	
qsrO	Fw	ATGCTTACGTTTTGGGCTAT	This study
	Rev	ATGGAAATGGATTCTTTTGAGTT	

Fig S1

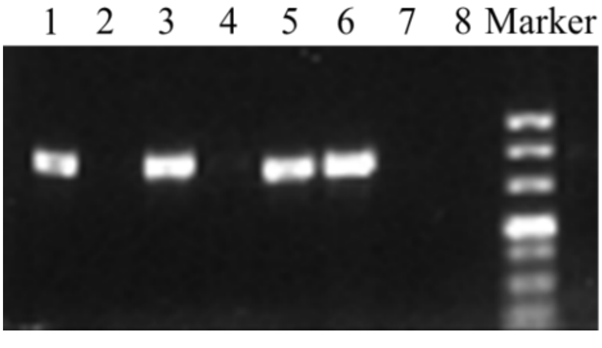

Fig S1 Agarose gel electrophoresis of *mvaT* and *mvaU* PCR products in PAO1 and the relevant knockout mutants. 1-4: Detection of *mvaT* gene, 5-8: Detection of *mvaU* gene. 1. PAO1, 2. PAO1ΔT, 3. PAO1ΔU, 4. PAO1ΔTΔU, 5. PAO1, 6. PAO1ΔT, 7.

PAO1ΔU, 8. PAO1ΔTΔU. Marker: DL500 (500 bp, 400 bp, 300 bp, 200 bp, 150 bp, 100 bp, 50 bp).

Fig S2

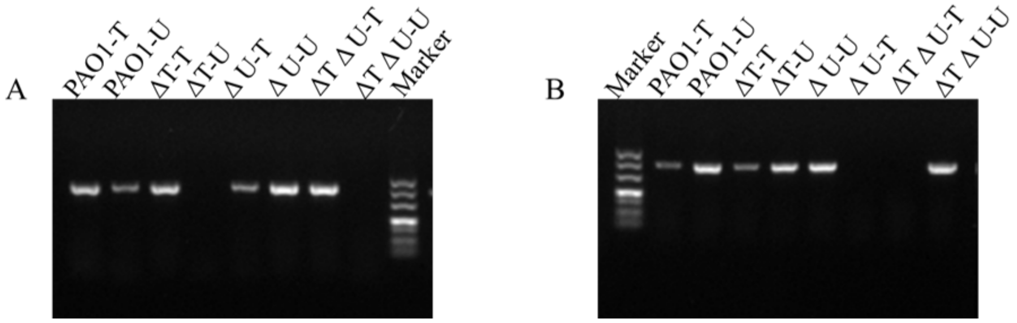

Fig S2 Agarose gel electrophoresis of *mvaT* and *mvaU* PCR products in PAO1 and the knockout mutants after plasmid complementation. A: Agarose gel electrophoresis images of *mvaT*. B: Agarose gel electrophoresis images of *mvaU*. Marker: 500 bp, 400 bp, 300 bp, 200 bp, 150 bp, 100 bp, 50 bp.

Fig S3

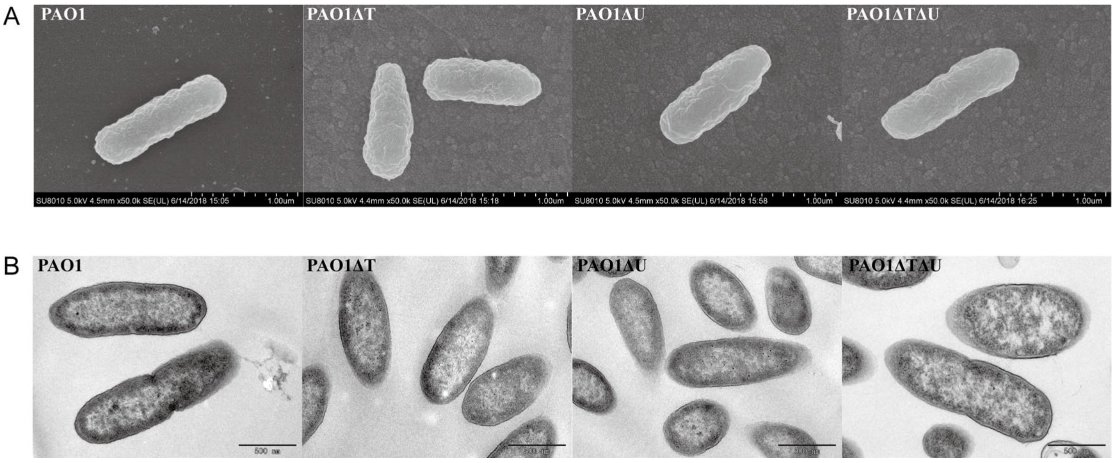

Fig S3 Images from SEM (A) and TEM (B) for PAO1 wild type and the *mvaT mvaU* knockout mutants. 1 mL of overnight cultures were centrifuged at 6000×g for 3 min and supernatants were discarded. Bacterial pellets were resuspended and washed in 1 mL 2.5% glutaraldehyde in phosphate-buffered saline (PBS). Tubes were fixed overnight at 4°C and fixatives were removed by centrifuged again at 6000×g for 3 min.

SEM was conducted by using a scanning electron microscope (FEI Quanta 2000), and TEM was conducted by using a transmission electron microscope (JEOL JEM-1200EX).

Fig S4

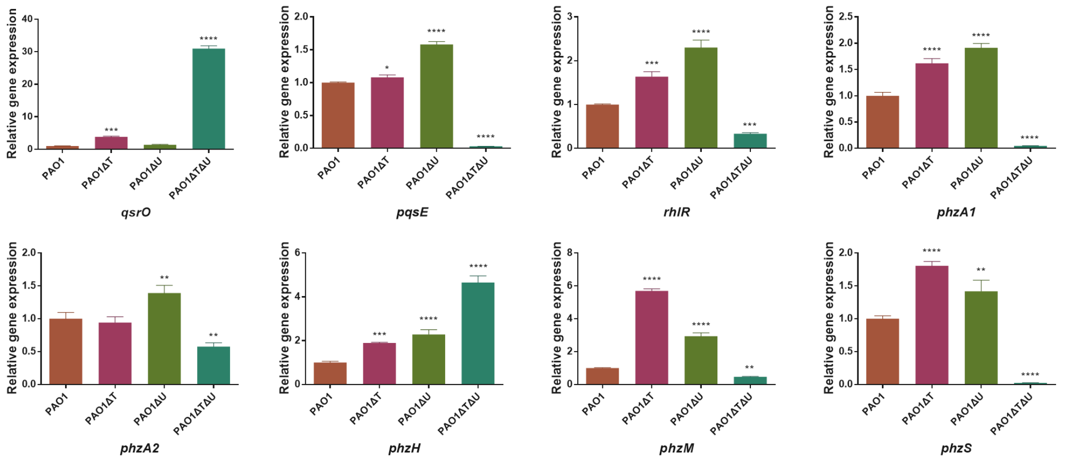

Fig S4. Effect of *mvaT mvaU* knockout mutation on representative gene expression in early stationary phase detected by Real -Time PCR. Data was calculated with one-way ANOVA and Bonferroni's multiple comparisons, in comparison with *P. aeruginosa* PAO1, *$P < 0.05$, **$P < 0.01$, ***$P < 0.001$, ****$P < 0.0001$.

Reference

1. Itoh Y. 1997. Cloning and characterization of the aru genes encoding enzymes of the catabolic arginine succinyltransferase pathway in Pseudomonas aeruginosa. J Bacteriol 179:7280-90.

2. Li C, Wally H, Miller SJ, Lu CD. 2009. The multifaceted proteins MvaT and MvaU, members of the H-NS family, control arginine metabolism, pyocyanin synthesis, and prophage activation in Pseudomonas aeruginosa PAO1. J Bacteriol 191:6211-8.

3. Schweizer HP. 1991. Escherichia-Pseudomonas shuttle vectors derived from

pUC18/19. Gene 97:109-21.

4. El-Mowafy SA, Abd El Galil KH, El-Messery SM, Shaaban MI. 2014. Aspirin is an efficient inhibitor of quorum sensing, virulence and toxins in Pseudomonas aeruginosa. Microb Pathog 74:25-32.

5. Viducic D, Murakami K, Amoh T, Ono T, Miyake Y. 2016. RpoN Modulates Carbapenem Tolerance in Pseudomonas aeruginosa through Pseudomonas Quinolone Signal and PqsE. Antimicrob Agents Chemother 60:5752-64.
